# Pulmonary Coccidioidomycosis in a Ruxolitinib-Treated Polycythemia Vera Patient: A Case Study and Literature Review

**DOI:** 10.7759/cureus.81725

**Published:** 2025-04-04

**Authors:** Angela M Zou, Blessing Eze, Abigail D'Souza, Quinta Mbah, Brandon Walls, Julien Bourgeois, Romain Rabany, Nancy Rolfe

**Affiliations:** 1 Internal Medicine, Creighton University School of Medicine, St. Joseph's Hospital and Medical Center, Phoenix, USA

**Keywords:** fluconazole, polycythemia vera, pulmonary coccidioidomycosis, ruxolitinib, ruxolitinib discontinuation syndrome

## Abstract

This case report explores the epidemiology and clinical implications of coccidioidomycosis (Valley fever), with a focus on cases involving the immunosuppressive agent ruxolitinib (RUX) in patients with polycythemia vera (PV). The incidence of coccidioidomycosis has increased in the past decade in endemic regions, particularly in immunocompromised individuals. RUX, a Janus-associated kinase 1/2 (JAK1/2) inhibitor used in PV treatment, has been associated with various infections, but its link to coccidioidomycosis remains underexplored. We present a rare case of coccidioidomycosis in a PV patient receiving RUX, highlighting the potential risks associated with this therapy. Our analysis suggests a possible increased risk of coccidioidomycosis in RUX-treated patients, especially in endemic areas, which is supported by retrospective cohort data. This case underscores the importance of heightened vigilance and consideration of prophylactic measures in patients receiving RUX, particularly in regions where coccidioidomycosis is prevalent. Understanding these associations can inform clinical management strategies and improve patient outcomes.

## Introduction

Coccidioidomycosis, or "San Joaquin Valley fever," is a systemic fungal infection contracted from the inhalation of Coccidioides immitis or Coccidioides posadasii spores found in soil. It is endemic to the southwestern U.S., northern Mexico, and parts of Central and South America, with cases reported as far north as Washington State [1,2]. Coccidioidomycosis incidence in the United States (U.S.) has been rising steadily over the past decade, from 8,232 new cases reported to the Centers for Disease Control and Prevention (CDC) in 2014 to 17,612 new cases reported in 2022 [3]. Over 95% of U.S. cases of coccidioidomycosis are from Arizona and California, particularly California’s Central Valley and southern Arizona. The true incidence is 6 to 14 times higher than that reported for misdiagnosed, unreported, untreated, or asymptomatic cases [4].

The risk of coccidioidomycosis is greater in those with high dust exposure, such as agricultural and construction workers and military personnel. Higher incidence rates are also observed in older adults (65+ in Arizona), men, pregnant individuals, and those who are immunosuppressed due to HIV (human immunodeficiency virus), hematologic malignancies, immunosuppressive agents, or organ transplants [5-9]. Furthermore, of the estimated 150,000 annual cases of coccidioidomycosis, approximately 40% are symptomatic and typically present with self-limited pulmonary disease [4]. Clinical presentation can range from mild "flu-like" illness to progressive pneumonia with symptoms including cough, fever, pleuritic pain, fatigue, and headaches, as well as transient skin manifestations including erythema nodosum [9]. A small percentage (5-10%) develop chronic pulmonary complications, and 1-2% develop disseminated disease [4]. 

In individuals with immunosuppression, rates of severe and disseminated infection are notably higher. While definitive incidence data are limited, associations have been identified linking increased rates of dissemination, mortality, and potential reactivation of latent coccidioidomycosis in solid-organ and allogeneic hematopoietic cell transplantation patients [10,11]. Certain biological response modifiers (BRMs), notably TNF-alpha inhibitors and inhibitors of T-cell activation, have been linked to instances of coccidioidomycosis [12,13]. These medications, while effective in modulating immune responses for various conditions, can inadvertently increase susceptibility to fungal infections such as coccidioidomycosis. Current treatment guidelines for individuals receiving BRMs advise screening with Coccidioides serology before starting BRMs in endemic regions, followed by regular monitoring for new symptoms [14]. However, routine serologic screening or antifungal prophylaxis for asymptomatic patients is not recommended [14].

For individuals on BRMs diagnosed with active coccidioidomycosis infection, oral azole therapy is typically recommended, with fluconazole doses ranging from 200 to 400 mg daily for 3-6 months or longer, particularly for uncomplicated cases of coccidioides pneumonia [14]. Treatment cessation is advised upon the resolution of symptoms, normalization of inflammatory markers, and stabilization of serologic and radiographic findings [14]. In cases of refractory disease or severe disseminated infection affecting soft tissues, bones/joints, or the meninges, amphotericin B is the recommended treatment [14]. This antifungal medication is employed when azole therapy is ineffective or when the infection becomes more severe, aiming to combat fungal infection and prevent further complications.

Ruxolitinib (RUX) is a medication classified as a Janus-associated kinase 1/2 (JAK1/2) inhibitor. It is approved as a second-line therapy for polycythemia vera (PV) in patients who do not respond well to or cannot tolerate hydroxyurea. This drug operates by modulating the JAK-STAT pathway, a crucial signaling pathway involved in cell growth, differentiation, and the immune response [15]. By targeting this pathway, RUX exerts an immunosuppressive effect, primarily by reducing cytokine signaling. This leads to the downregulation of both innate and adaptive immune system components [15-17]. The use of RUX has been associated with an increased risk of varicella-zoster virus (VZV) infection, but the extent of risk for other infections, including fungal infections, has not been precisely determined [16]. Among fungal infections linked to RUX, cryptococcal infections, both pulmonary and extrapulmonary, have been the most reported [16,18]. However, cases of RUX-associated Aspergillus, Candida, and Pneumocystis jiroveci infections have also been documented. Notably, data regarding patients receiving RUX therapy, specifically for the treatment of PV, are limited. Through our comprehensive literature review, we found a few instances of coccidioidomycosis in patients with PV receiving RUX. These few reported cases suggest a significant gap in understanding the potential risks and interactions between RUX therapy and fungal infections such as coccidioidomycosis in PV patients. Further research is warranted to elucidate the relationships among RUX treatment, PV management, and susceptibility to fungal infections in this patient population.

## Case presentation

A 50-year-old Caucasian man with PV who was receiving treatment with RUX presented to an emergency department (ED) in Arizona. He reported one day of constant severe chest discomfort following one month of worsening shortness of breath and a nonproductive cough. Additionally, he had experienced intermittent fevers with chills, night sweats, and headaches throughout the same month. He had visited his primary care physician (PCP) a week prior to ED presentation and was advised to try symptomatic treatment with acetaminophen and dextromethorphan, which did not resolve his symptoms. Suspecting pneumonia, his PCP also began an infectious workup including coccidioides antibody testing (IgM and IgG) and called to inform him of a positive coccidioidomycosis result shortly after he arrived at the ED. Due to the severity of his presentation and complex medical history necessitating specialized services, he was then transferred to our facility for higher-level care.

The patient had been diagnosed with JAK2-positive PV five years prior, initially presenting with fatigue, headaches, diffuse myalgias, fifteen-pound weight loss, night sweats, aquagenic pruritus, and hepatosplenomegaly. Despite being treated with weekly phlebotomies and high-dose hydroxyurea (titrated up to 2000 mg daily), the patient experienced continued symptoms and clinical deterioration, including massive splenomegaly and thrombotic events. He subsequently commenced RUX therapy at 10 mg twice daily, which helped stabilize his blood cell counts and resolved most of his symptoms, including splenomegaly. During the next two years, his RUX dosage was gradually increased to 25 mg twice daily due to continued symptoms of fatigue and bone pain. At the time of admission, the patient had been on RUX 25 mg twice daily for three years, prophylactic acyclovir 800 mg daily for herpes zoster prevention following a previous outbreak one year prior, and aspirin 81 mg daily. While most of his PV symptoms were controlled, he still experienced chronic fatigue and required phlebotomy approximately once a year since starting RUX. Routine bloodwork from six months before admission showed a normal white blood cell count of 7.8 x 10^3μL, a normal hemoglobin level of 15 g/dL, and a normal platelet count of 339 x 10^3/μL. He had no other medical conditions or history of infections other than herpes zoster while on RUX. He relocated to Arizona three years ago from Oregon and was working in construction at the time of admission. On admission to our facility, the patient presented with a temperature of 37.8°C, a heart rate of 112 bpm, and an elevated blood pressure of 140/90 mmHg. His oxygen saturation was initially 96% on pulse oximetry while breathing room air but decreased to 88% after an hour, at which point he was placed on 2 L/min of supplemental oxygen via a nasal cannula with improvement to 94%. He continued to require 1-2 L/min of supplemental oxygen for the next five days in order to maintain oxygen saturation above 92%. The physical examination revealed an ill-appearing and diaphoretic patient with dry mucous membranes. Chest auscultation revealed mildly diminished breath sounds bilaterally with normal respiratory excursion.

Initial laboratory findings revealed leukocytosis with a white blood cell count of 16.1 × 10^3/μL (neutrophils 76%, lymphocytes 9%), a hemoglobin level of 12.6 gm/dL, a hematocrit of 37%, and a platelet count of 438 × 10^3/μL. The D-dimer level was elevated at 1.97 mg/L, the prothrombin time (PT) was 15.3 seconds, and the procalcitonin level was within normal limits. Chest X-ray revealed a possible left perihilar mass or adenopathy, prompting further evaluation with a computed tomography (CT) scan.

Microbiology and serology studies revealed positive Cocci IgG (5.5) and IgM (5.5), a complement fixation titer of 1:8 for Coccidioides antibodies, and positive detection of Coccidioides immitis antibodies by immunodiffusion, indicating current or recent infection. QuantiFERON TB Gold Plus testing and PCR testing of a nasopharyngeal swab for influenza A/B, COVID-19, and respiratory syncytial virus (RSV) were negative. HIV1/2 Ag/Ab testing returned negative results. Sputum cultures grew normal respiratory flora along with light growth of Coccidioides immitis/posadasii. Blood cultures taken at admission revealed no growth.

**Figure 1:**
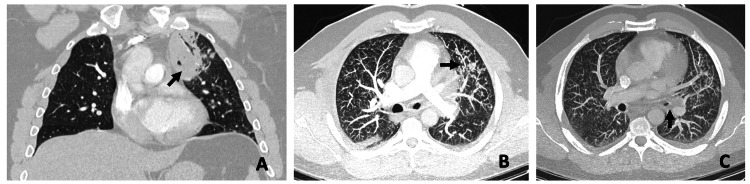
Computed tomography angiography (CTA) images of patient's chest. (A) Mass-like opacity with air bronchograms in medial left upper lung lobe with (B) multiple bronchocentric tree-in-bud nodules in the left upper lobe​ and (C) left hilar lymphadenopathy

Computed tomography angiography (CTA) with contrast of the chest revealed a mass-like opacity with air bronchograms in the medial left upper lobe, accompanied by multiple tree-in-bud nodules. Additionally, both posterior lower lobes exhibited subpleural interstitial ground‒glass opacities with trace pleural effusions bilaterally. Enlarged lymph nodes were observed in the mediastinum and left hilum, with the largest node measuring 2.4 cm (Figure 1). These findings were most consistent with pneumonia secondary to pulmonary coccidiomycosis, with a low suspicion of malignancy.

The medical team, patient, family, and oncologist deliberated on potential interactions between fluconazole and RUX. Given the severe pulmonary infection, infectious disease specialists advised a high dose of 400 mg fluconazole daily for at least one year. To prevent RUX toxicity, a gradual reduction in the RUX dose from 25 mg twice daily to 7.5 mg twice daily was initiated, alongside a gradual increase in fluconazole from 200 mg to 400 mg daily over several days. The patient was given 10 mg of RUX twice daily with 200 mg of fluconazole daily for five days, which was then adjusted to RUX 7.5 mg twice daily with fluconazole 400 mg daily to be continued after discharge three days later. This approach aimed to address the infection effectively while minimizing adverse effects. The patient's previous experience of PV symptom recurrence after missing RUX doses and concern for RUX-discontinuation syndrome (RDS) guided the decision to maintain an RUX regimen. Hematology/Oncology endorsed this strategy, emphasizing close monitoring for potential adverse effects to ensure patient safety and treatment efficacy.

The patient continued to have temperatures of 38°C and above daily and increased dry cough for six days after admission despite starting antifungal therapy, prompting further investigation to rule out coinfection or malignancy. Flexible bronchoscopy with bronchoalveolar lavage (BAL) of the left upper lobe mass, transbronchial biopsies of the left hilar mass, and endobronchial ultrasound-guided transbronchial needle aspiration of multiple lymph nodes were performed. BAL cultures grew Coccidioides immitis/posadasii. Cultures from BAL and lung biopsies were negative for anaerobic organisms, Nocardia/Actinomyces, acid-fast bacilli, and Pneumocystis. Lymph node 7 (subcarinal location) biopsy revealed no granulomas or malignant cells, whereas lymph node 10 L (hilar location) biopsy revealed rare fungal spores identified by Grocott-Methenamine Silver (GMS) staining. Biopsies of the remaining lymph node and soft tissue hilar mass revealed benign bronchial cells.

Concurrently, the patient developed new subcutaneous skin nodules on the left upper and right lower extremities a few days after admission, raising suspicion of erythema nodosum or disseminated coccidioidomycosis. A punch biopsy of the left upper extremity nodule revealed benign fibrofatty tissue without granulomatous inflammation or fungal evidence. GMS staining of the biopsy was negative for fungal organisms, indicating an absence of fungal infection in the sampled tissue. Consequently, these soft tissue nodules were considered to be consistent with erythema nodosum. 

Having ruled out malignancy or coinfection, the patient was continued on his antifungal regimen with the increase of fluconazole from 200 to 400 mg five days into his admission. The patient's fever and chills gradually subsided, and his cough significantly improved after the bronchoscopy. His skin nodules had not resolved by the time of discharge. The patient was successfully weaned off supplemental oxygen a week into his admission, and he was discharged after eight days total in the hospital. Discharge medications included RUX 7.5 mg twice daily, fluconazole 400 mg daily, and benzonatate, guaifenesin, and robaxin for symptomatic cough relief. Planned duration of antifungal treatment was at least one year with adjustment based on resolution of symptoms and laboratory monitoring. Follow-up appointments were scheduled with the Pulmonology, Infectious Disease, and Hematology/Oncology clinics for potential RUX dosage adjustments and repeat CT scans to monitor pulmonary coccidioidomycosis. One month after discharge, a hematology/oncology evaluation revealed a stable white blood cell count (9.1 × 10^3/μL), hemoglobin (12.4 gm/dL), hematocrit (39.3%), and platelet count (381 × 10^3/μL). The patient is currently being closely monitored with bloodwork for RUX toxicity and cocci serologies to track infection resolution.

## Discussion

The occurrence of pulmonary coccidioidomycosis in a PV patient receiving RUX alone is noteworthy. While an increased risk of VZV infection has been observed, the literature suggests similar infection rates in RUX-treated patients and controls. However, individual case reports have associated RUX with various infections, including toxoplasmosis retinitis, disseminated molluscum contagiosum, Mycobacterium tuberculosis infection, and hepatitis B reactivation in myelofibrosis patients [19,20]. Additionally, fungal infections in RUX users have been sporadically reported, with Cryptococcus, Aspergillosis, and Candida being the most common [16,21]. However, reports of RUX-associated coccidioidomycosis infections remain limited.

In a retrospective cohort study involving 135 patients receiving RUX treatment in regions endemic to Coccidioides, researchers observed a small subset (3%) of patients who were diagnosed with coccidioidomycosis [22]. This infection was found in patients with conditions such as myelofibrosis and graft-versus-host disease who were taking RUX dosages ranging from 5 to 20 mg twice daily. Notably, among these cases, instances of disseminated disease were identified and occurred more frequently in patients not prescribed prophylactic antifungal medications or concurrently using additional immunosuppressive agents [22]. This highlights the importance of considering prophylactic measures and careful monitoring in RUX-treated patients, particularly in areas where fungal infections such as coccidioidomycosis are prevalent. In the present case, the patient was solely treated with RUX therapy and prophylactic acyclovir, suggesting a potential elevated risk of coccidioidomycosis infection attributed solely to RUX administration. In addition to prolonged exposure to a high dose of RUX, other risk factors were identified in our patient’s case, including the occupational exposure related to his construction work. This underscores the importance of recognizing both medication-related risks and environmental factors in assessing the susceptibility of patients to endemic infections such as coccidioidomycosis.

The simultaneous administration of oral azoles for coccidioidomycosis alongside ongoing RUX treatment presents complexities in treatment strategies. RUX is metabolized by hepatic cytochrome P450 enzymes, particularly CYP3A4 and CYP2C9, which are inhibited by azoles [23]. For example, fluconazole moderately inhibits both CYP3A4 and CYP2C9, exerting a more substantial effect on RUX pharmacokinetics than a single strong pathway inhibitor, such as ketoconazole, on CYP3A4 [24]. This interaction underscores the need for careful consideration and management of drug regimens to optimize therapeutic outcomes while minimizing adverse effects.

Elevated serum levels of RUX pose a heightened risk of adverse effects, including thrombocytopenia, anemia, neutropenia, and subsequent susceptibility to infection [25]. Moreover, fluconazole dosages ranging from 200 to 400 mg per day have demonstrated tolerability and efficacy in treating chronic pulmonary or nonmeningeal disseminated coccidioidomycosis, establishing them as standard treatments [26]. Treatment duration with fluconazole depends on response to therapy and is often longer than one year with high rates of symptom recurrence after treatment cessation that require retreatment [26]. However, caution is advised when co-administering fluconazole with RUX, especially at doses exceeding 200 mg daily, as emphasized by Food and Drug Administration (FDA) warnings [25]. This underscores the importance of vigilant monitoring and dosage adjustments to minimize potential adverse interactions.

Concurrently, concerns about RDS and withdrawal-related complications arose in our patient. His prior experience of symptom recurrence after missing an RUX dose and the presence of splenomegaly before initiating RUX heightened these concerns. RDS entails an acute relapse of disease symptoms upon abrupt discontinuation of RUX, posing risks for serious adverse effects. These may include the exacerbation of splenomegaly, cytopenia, respiratory distress, and hemodynamic instability, potentially leading to conditions resembling septic shock or disseminated intravascular coagulation syndromes [27,28]. RDS may affect approximately 15% of patients following RUX cessation, with an increased risk observed in those with substantial disease burden and features such as large splenomegaly [29]. Some case reports indicate that RDS typically manifests within three weeks of RUX discontinuation, irrespective of the RUX dose, often necessitating RUX reintroduction followed by gradual tapering [27]. Thus, abrupt discontinuation of RUX is strongly advised against to mitigate the risk of RDS and its associated complications.

Clinical guidance for managing coccidioidomycosis alongside RUX administration is presently insufficient. A prior study revealed that while co-administration of RUX with multiple doses of fluconazole (200 mg for seven days) notably elevated RUX systemic exposure in healthy individuals, no adverse events linked to RUX occurred in the same study [22]. This finding indicates that concurrent use of RUX with fluconazole may necessitate dose adjustments but not discontinuation. In our patient's case, collaborative decision-making involving infectious disease specialists and hematologists resulted in a tailored approach. This involved temporarily reducing the RUX dosage while escalating fluconazole. Despite initial concerns about symptomatic relapse upon RUX discontinuation, the patient's adherence to the adjusted regimen led to significant and sustained improvement in pulmonary symptoms. Continuous monitoring and adjustment of the RUX dosage alongside fluconazole administration are imperative for optimizing therapeutic outcomes while managing coccidioidomycosis infection.

Approaches to this dilemma in reported cases vary, with some opting for RUX discontinuation, dose reduction, or maintaining unchanged RUX dosing during fluconazole therapy. However, concerns regarding potential compromises in antifungal efficacy, particularly in fungal infections such as coccidioidomycosis, highlight the necessity for careful consideration. It is essential to emphasize that more studies are needed to better understand the optimal management strategies in such cases and to elucidate the potential impact on treatment outcomes.

## Conclusions

In conclusion, the management of coccidioidomycosis in patients undergoing treatment with RUX presents a complex dilemma, with varying approaches reported in the literature. While some advocate for RUX discontinuation, dose reduction, or unchanged dosing alongside fluconazole therapy, concerns persist regarding potential compromises in efficacy, especially in fungal infections such as coccidioidomycosis. Collaborative decision-making involving infectious disease specialists and hematologists is crucial in tailoring treatment strategies. However, it is imperative to acknowledge the need for further research to establish optimal management approaches and their impact on treatment outcomes. Ongoing monitoring and adjustment of the RUX dosage alongside fluconazole administration remain essential for optimizing therapeutic outcomes while managing coccidioidomycosis infection.
